# The Impact of the Deepwater Horizon Oil Spill upon Lung Health—Mouse Model-Based RNA-Seq Analyses †

**DOI:** 10.3390/ijerph17155466

**Published:** 2020-07-29

**Authors:** Yao-Zhong Liu, Charles A Miller, Yan Zhuang, Sudurika S Mukhopadhyay, Shigeki Saito, Edward B. Overton, Gilbert F Morris

**Affiliations:** 1 Department of Biostatistics and Data Science, Tulane University School of Public Health and Tropical Medicine, New Orleans, LA 70112, USA; 2 Department of Environmental Health Sciences, Tulane University School of Public Health and Tropical Medicine, New Orleans, LA 70112, USA; rellim@tulane.edu; 3 Division of Pulmonary, Critical Care and Environmental Medicine, Department of Internal Medicine, Tulane University School of Medicine, New Orleans, LA 70112, USA; yzhuang1@tulane.edu (Y.Z.); ssaito@tulane.edu (S.S.); 4 Department of Pathology and Laboratory Medicine, Tulane University School of Medicine, New Orleans, LA 70112, USA; smukhopa@tulane.edu (S.S.M.); gmorris2@tulane.edu (G.F.M.); 5 Environmental Sciences Department, Louisiana State University, Baton Rouge, LA 70112, USA; ebovert@lsu.edu

**Keywords:** BP oil spill, RNA-seq, lung cancer, dispersant, Corexit 9527, DNA damage, lung inflammation, K-Ras^LA1^ mice

## Abstract

We used a transcriptomic approach to interrogate the effects of a saline-accommodated fraction from the Macondo 252 well (MC252) oil and Corexit dispersants on lung tissue. Wild-type C57BL/6 male and female mice were exposed on days 0, 7 and 13 by oropharyngeal aspiration to saline accommodated fractions (SAF) of crude oil from the Macondo (MC252) well, Corexit 9500, Corexit 9527, 9500+oil and 9527+oil or a saline solution as the vehicle control. These treatments did not cause overt toxicity, with the exception of the Corexit exposures which caused brief weight loss after the first exposure. On day 14, total RNA was isolated from the left lung for RNA-seq analyses. KEGG-pathway-based differential expression revealed that Corexit 9527 elicited the strongest changes involving the upregulation of 19 KEGG pathways (FDR < 0.10), followed by Corexit 9500 with the upregulation of seven pathways (FDR < 0.10). As an important signature, pathways related to a response to DNA damage (e.g., p53 signaling and mismatch repair) dominate those upregulated by Corexit 9527 and Corexit 9500. In addition, pro-inflammatory pathways (e.g., cytokine-cytokine receptor interaction, IL-17 signaling pathway and TNF signaling pathways) were upregulated selectively in oil-treated male mice. Surprisingly, oil + dispersant combinations caused lesser effects than the individual treatments at the transcriptomic level. Overall, these findings support potential genotoxicity, inflammation and cell death due to dispersant or oil exposures. Similar exposures to lung tumor bearing K-Ras^LA1^ mice provided evidence for tumor promotion by oil and Corexit dispersant treatments. Our mouse RNA-seq analyses may be relevant to the pulmonary health hazards of MC252 oil and dispersants experienced in exposed populations.

## 1. Introduction 

The Deepwater Horizon oil spill (the BP oil spill) was the largest marine oil spill in the history of the petroleum industry and one of the largest environmental disasters in American history [1,2]. During the period of ~5 months (from the explosion of Deepwater Horizon on 20 April 2010 to 19 September 2010), when the well was formally declared sealed, approximately 210 million US gallons of crude oil spilled [1,2] and 1.84 million US gallons of oil dispersants were applied [3,4]. Approximately 50,000 workers were involved in the cleanup during and after the spill. The formation of aerosols containing oil and dispersants on the sea surface at the site of oil spill [5] could present a public health threat. Consequently, short- and long-term health effects might occur from inhaling oil and dispersant chemicals. Respiratory irritation symptoms have been reported in both cleanup workers [6] and the local women of southern Louisiana following the oil spill [7]. Evidence of genotoxic effects that persisted 5–6 years after the Prestige oil spill incident have been reported [8,9]. Therefore, performing experiments under controlled environmental conditions may increase our understanding of the effects on people years after the exposure.

Corexit 9527 [10] and 9500 [10] were the principal dispersants used on the oil that spilled from the Macondo 252 well (MC252, Deepwater Horizon/BP oil spill). There is some evidence that 2-butoxy ethanol, a principal component of Corexit 9527, may be carcinogenic. In a long-term carcinogenesis study, pulmonary exposures to 2-butoxy ethanol produced sex-specific toxicity and cancers of the adrenal glands, forestomach, and liver after chronic pulmonary exposures at relatively high treatments in mice but not rats [11]. The tumorigenic potential of Corexit 9500 or its components have not been described. Oil from the Macondo 252 (MC252) well is a highly characterized light, sweet crude. Crude oils are generally known for their genotoxicity and carcinogenic effects as they contain a multitude of toxic chemicals [12].

Our previous RNA seq studies [13,14] of exposed human airway epithelial cells (BEAS-2B cells) identified five KEGG pathways upregulated by Corexit 9527, including ribosomal biosynthesis (hsa03008) (*p* = 1.97 × 10^−13^), protein-processing (hsa04141) (*p* = 4.09 × 10^−7^), Wnt signaling (hsa04310) (*p* = 6.76 × 10^−3^), neurotrophin signaling (hsa04722) (*p* = 7.73 × 10^−3^) and insulin signaling (hsa04910) (*p* = 1.16 × 10^−2^) pathways. The co-upregulation of the above pathways suggest carcinogenic potential for Corexit 9527 [15,16,17]. Here, we extended the previous cell culture studies [13,14] to a murine model. We conducted experiments using the wild-type C57BL/6 (B6) mice of both sexes treated with saline-accommodated fractions (SAF) of MC252 crude oil (treatment group 1), Corexit 9500 (treatment group 2), Corexit 9527 (treatment group 3), 9500+oil (treatment group 4), 9527+oil (treatment group 5) and saline (as controls) (treatment group 6). The lung samples of the treated mice were used for RNA-seq analysis, qRT-PCR, and histology. To assess whether these treatments had effects on tumorigenesis, we also exposed lung tumor bearing K-ras^LA1^ mice in a similar manner to the B6 mice. Lung tumorigenesis indices, including pleural surface tumor count, tumor burden and tumor cell numbers, were used to compare the effects between a treatment and control (saline).

The most pronounced effects in our previous [14] and present study were observed for the treatment with the SAF of Corexit 9527, which was highlighted by upregulation of a number of pathways related to DNA damage. Similarly, some of the DNA damage pathways were also upregulated by the Corexit 9500 SAF. Moreover, proinflammatory pathways were upregulated by the crude oil SAF in male mice. Consistent with the above observations at the transcriptomics level, the three treatments also increased promotion of lung tumors in the K-ras^LA1^ mice. Overall, our findings provide evidence supporting DNA damage, proliferation, inflammation, and tumor promotion of oil-spill chemicals for the pulmonary system.

## 2. Methods

### 2.1. Preparation of Saline Accomodated Fraction (SAF)

Louisiana Sweet Crude Oil was kindly provided by The Architecture, Engineering, Consulting, Operations and Management Company (AECOM, Los Angeles, CA, USA). This oil was obtained from the site of the Macondo well during the BP Oil Spill disaster. Many studies on the composition and effects of MC252 oil are reported at the Gulf of Mexico Research Initiative’s GRIIDC database (https://data.gulfresearchinitiative.org). Commercially available Corexit EC9500A and EC9527 dispersants were kindly provided by a contract between Nalco/Exxon Energy Chemicals, L.P. (Sugar Land, TX, USA) and Tulane University (New Orleans, USA). The dispersants are liquid solutions ready for use. Corexit 9527 is a commercial grade mixture of propylene glycol (1–5%), 2-butoxy ethanol (30.0–60.0%), and proprietary organic sulfonic acid salts (10–30%) [10]. Corexit 9500 consists of dioctyl sodium sulfosuccinate (DOSS, 18.2%), sorbitan and isosorbide containing polyethoxylates (2.8%), monoesters (15.8%), diesters (23.9%), triesters (17.3%) and tetraesters (3.3%) and the remaining18.7% is dipropylene glycol butyl ether (DGBE) and Norpar12 [10], a mixture of paraffins that are 5 to 20 carbons in length.

Saline-accommodated fractions (SAF) of MC252 oil, Corexit 9500, Corexit 9527, 9500+oil, and 9527+oil were made per the protocol in our previous study with the exception that Hank’s Balanced Salt Solution (HBSS) replaced water [13]. Briefly, fractions containing oil were prepared at a 1 part oil to 20 parts HBSS ratio, Corexit solutions were prepared at 1:40 HBSS ratios, and the combinations had 0.5 parts Corexit +1 part oil to 20 parts HBSS. After mixing vigorously for 24 h at 300 rpm, the solutions were allowed to separate and the saline phase (for the oil alone sample) was removed and used for the exposures. The other mixtures did not readily phase separate and were used directly (Figure S1).

### 2.2. Treatment of Wild-Type C57BL/6 Mice for Transcriptome Analysis

Animal protocols were reviewed by the Tulane University institutional animal care and use committee (Protocol ID 106: Impact of Oil Spill to Human Lung Health—Next Generation Sequence and Mouse Model Based Analyses. Date of approval: 02/26/2018). Wild-type C57BL/6 mice 7 weeks of age were purchased from Charles River and acclimated to the vivarium for 1 week before commencing the experiment. The mice were maintained under specific pathogen free conditions and provided food and water ad libitum. Six treatment groups, i.e., oil, Corexit 9500, Corexit 9527, 9500+oil, 9527+oil, saline (control), were set up, with each group containing 3 male or 3 female mice. After 10-fold dilution in HBSS (0.1 SAF), 50µl of each 0.1 SAF was delivered to mice by oropharyngeal aspiration as previously described [18]. Treatments with 0.1 SAFs were on days 0, 7 and 13, with euthanasia and tissue collection on day 14. Upon harvest, the left lung was placed in 1ml Trizol (Ambion) and homogenized in a bead mill homogenizer at power 5 for 60 s. The homogenate was frozen at −80 °C for processing later. The right lung was fixed by perfusion with formalin for histological analyses as previously described [19].

### 2.3. Treatment of K-Ras^LA1^ Mice

Heterozygous K-Ras^LA1^ mice in the C57BL/6 background were provided by Dr. Tyler Jacks through the National Cancer Institute Mouse Repository. These mice, which harbor a duplication of exon 1 in a non-functional allele of K-Ras, spontaneously develop multi-focal lung tumors by 6-8 weeks of age due to stochastic homologous recombination [20].

Male (3–4 per treatment group) and female K-ras^LA1^ 8 week old mice (3–4 per treatment group) were treated by oropharyngeal aspiration with 50µL 0.1 SAFs of oil, 9500, 9527, 9500+oil, 9527+oil and saline as described above for wild-type B6 mice on days 0, 7, 14 and euthanized at day 21. The lung tissue was processed as described above and the fixed right lung was characterized for lung tumorigenesis indices, i.e., pleural surface tumor count, lung tumor burden, lung tumor cell count. In total, there were 42 mice, including 4 mice of each sex in both the saline (control) and oil groups, 4 male mice in the 9527 and the 9527+oil groups and 3 mice of each sex in each of the remaining treatment groups.

### 2.4. RNA-seq of Wild-Type C57BL/6 Mice

The Trizol homogenate was thawed to room temperature and 200 µl chloroform was added followed by vortexing. The mixture was centrifuged at 12,000× *g* for 15 min at 4 °C in a heavy phase lock gel tube (Five Prime). The upper phase was transferred to a tube containing an equal volume of 70% ethanol. Subsequent RNA purification was with a RNeasy mini kit according to the manufacturer’s specifications (Qiagen, Germantown, MD, USA). DNA was removed from the isolated RNA by DNase I treatment (Qiagen, Germantown, MD, USA) and the RNA was re-purified by RNeasy mini kit.

Total RNA was sent to Omega Bioservices (Norcross, GA, USA) for sequencing. At Omega Bioservices (Norcross, GA, USA), before RNA-sequencing, the total RNA for each specimen was checked for quality using Agilent TapeStation (Agilent Technologies, Santa Clara, CA, USA). The RNA Integrity Numbers (RINs) for all the specimens as obtained by TapeStation passed a score of 8.0 or greater. Upon quality check, mRNA sequencing was performed using the Illumina TruSeq Stranded mRNA sequencing kit. The sequencing depth was at least 40 million reads, with paired end reads of 150 bps (PE150). The raw RNA-seq data were submitted to NCBI GEO (Gene Expression Omnibus) with accession number of GSE137204.

### 2.5. Characterization of K-Ras^LA1^ Mice

For each K-Ras^LA1^ mouse, the number of pleural surface tumors on fixed lung tissue was counted by three investigators, who were blind to the treatment group assignment. The counts from three investigators were averaged to represent the number of pleural surface tumors for that specimen for downstream statistical analyses. Tissue sections prepared from paraffin-embedded fixed lung tissue were cut at 5µm and stained with hematoxylin and eosin (H&E) before evaluation of tumor burden and tumor cell number. The tumor burden (defined as the ratio of hyperplastic lesion area to total lung section area on H&E-stained sections) was quantified with an Aperio ScanScope slide scanner. Tumor cell number was the total number of cells contained in all the specific tumor areas as determined by the image analysis software (Genie) of the slide scanner.

### 2.6. RNA-seq Data Analysis

RNA-seq raw data (fastq data) passed for overall quality using FastQC program (https://www.bioinformatics.babraham.ac.uk/projects/fastqc/).The transcript quantification analysis of fastq data was performed using Salmon program [21]. Specifically, an index file was first built using the reference transcriptome for mouse (Mus_musculus.GRCm38.cdna.all.fa). Then, fastq data of each sample were aligned to the index file for transcript quantification using “salmon quant” command, which resulted in a quant.sf file containing count information for each transcript. The resultant files, quant.sf files, for all the samples were then grouped and analyzed together using R and Bioconductor’s tximport package [22], which generated a count matrix, with rows as transcript IDs and columns as specific samples.

The count matrix was then analyzed using DESeq2 package [23] for differential expression analysis, which produced differentially expressed genes between two comparison groups based on a model using the negative binomial distribution. To discover differential expression at the pathway level, another package, GAGE [24], was used to identify those KEGG pathways that are differentially regulated between two conditions. The package is based on a meta-test that summarizes t test statistics (for differential expression at the individual gene level) for all genes contained in a pathway. Differential pathway expression may be identified if a large number of individual genes in the pathway have the same direction of differential expression. An FDR value of <0.10 was used for significance threshold.

We submitted up- and downregulated gene expression data (*p* < 0.05) to DAVID [25] (https://david.ncifcrf.gov) to obtain functional annotation (e.g., GO terms). A Bonferroni corrected *p* value < 0.05 was used for significance threshold of gene set enrichment. The above differential expression analysis and gene set enrichment were performed on the total (pooled both sexes) sample and sex-specific samples respectively.

### 2.7. Real-Time PCR Experiments

RNA purity and concentration were measured using a NanoDrop spectrophotometer (Thermo Scientific). First-strand cDNA was generated by reverse transcription using the *iScript* cDNA Synthesis Kit (Bio-Rad, Hercules, CA, USA). Quantitative PCR was performed with primer sets shown in Supplemental Table S1 with the iQ SYBR Green Supermix (Bio-Rad). PCR conditions were 95 °C for 3 min, followed by 45 cycles at 95 °C for 15 s, 60 °C for 30 s, and 72 °C for 15 s. After PCR, a melting curve inferred the specificity of the amplification. Relative expression of a specific target mRNA was normalized against a mouse internal control mRNA, 36B4 (a ribosomal protein).

### 2.8. Statistical Analysis of Tumor Indices of K-Ras^LA1^ Mice and Real-Time PCR Data

An extreme outlier (mouse #17), which is defined as >3 IQR (interquartile range) from the median, was identified for tumor cell number and tumor burden and hence was removed for subsequent analysis.

We used a Poisson regression model (with *glm* function in R) to analyze the association of pleural surface tumor count with different treatments, while adjusting for sex. We used the *lm* function in R to analyze the association of log-transformed tumor burden and log-transformed tumor cell count with different treatments, while adjusting for sex. This adjustment of sex aimed to remove the difference in an outcome of interest (e.g., tumor burden) that might be attributed to the sex difference so as to achieve a more precise estimate of the treatment effects. We used a multiple regression analysis approach, as implemented in the *lm* function in R, to analyze the real-time PCR data, which follows the method by Yuan et al. [26]. As compared with the traditional delta–delta CT method, this method can accommodate covariate information (such as sex in our study) in the real-time PCR experimental design. Specifically, for each mouse subject, the CT values for the reference gene (36B4 gene) and the target gene (e.g., Chek1 gene) were averaged over technical replicates. Then the CT value of the reference gene was deducted from the CT of the target gene to obtain the delta CT value, i.e., the delta CT value was tested for effect of a treatment (e.g., Corexit 9527) while adjusting for sex under regression analysis, where we coded the treatment variable under two levels, e.g., Corexit 9527 and control, with control as the “baseline” level. The fold change of the treatment (Corexit 9527) vs. control (saline) was estimated through regression coefficient for the treatment term, i.e., 2 to the negative power of the regression coefficient. A significant regression coefficient for treatment variable indicates confirmation of real-time PCR results. For example, a significant (*p* < 0.05) “negative” regression coefficient of treatment, suggesting a lower delta CT value in the treatment, e.g., Corexit 9527, as compared to the control, confirms “upregulation” of a gene in the treatment vs. control.

## 3. Results

### 3.1. Pilot Experiments Determined Tolerable Treatments for RNA-seq Experiments

Our initial experiment with three female mice was designed to determine a dose of Corexit 9500+oil SAF that caused lung injury in mice. Previous experiments indicated that the Corexit 9500+oil and Corexit 9527+oil SAF displayed similar toxicity curves with BEAS-2B cells in culture and that at 1/100 dilution of both solutions was toxic [27]. Therefore, mice were treated by oropharyngeal aspiration with 50µL of the 1/100 dilution SAF and 10-fold higher (1/10) and lower dilutions (1/1,000) SAF to assess their tolerability. Mice sacrificed on day 14 tolerated three treatments (day 0, 7 and 13) with each dilution of Corexit 9500+oil. The mouse exposed to the 1/10 dilution of Corexit 9500+oil SAF displayed modest weight loss (<5%) on the initial treatment but recovered and did not exhibit significant weight loss on the two subsequent treatments. Moreover, H&E stained tissue sections of fixed lung tissue from the exposed mice showed modest evidence of lung injury. Subsequent experiments were performed with 1/10 dilution of each SAF.

A second preliminary experiment was performed with six female mice with one mouse treated with saline, oil, Corexit 9500, Corexit 9527, Corexit 9500+oil, Corexit 9527+oil, respectively, on day 0, 7 and 13 @ a concentration of 1/10 SAF before euthanasia on day 14. A third preliminary experiment was performed with male mice that repeated the second preliminary experiment.

For each experiment, each treatment regimen was tolerated. The mice treated with 1/10 SAF of Corexit 9500 or Corexit 9527 exhibited weight loss (~10%) on the initial treatment but recovered to gain weight through subsequent treatments. Surprisingly, the addition of oil to the SAF of the dispersant (either Corexit 9500 or Corexit 9527) reduced the severity of weight loss to ~2% on the initial treatment. Microscopic assessment of H&E stained sections of fixed lung tissue revealed areas of minor focal inflammation that were most pronounced in the mouse treated with Corexit 9527.

The gross and histological assessments of female and male mice in the second and third preliminary experiments are presented in Supplemental Figure S3. Additionally presented is the weight change data in second preliminary experiment.

### 3.2. Total Sample Analyses Identified DNA Damage Effects from Corexit 9527/9500

**Figure 1:**
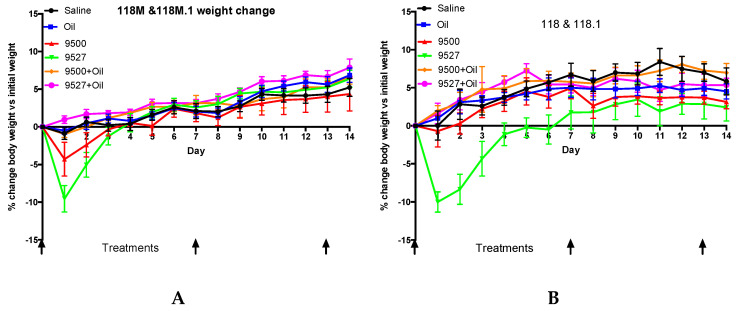
Two-week weight changes of mice relative to treatments. (**A**) is for male and (**B**) is for female mice, with *n* = 6 for each sex and treatment. Error bars shown are standard error.

We observed a stable weight increase in the treated mice over the two weeks with the exception of the Corexit 9527 treatment, which induced a drop of weight on the initial few days in both male and female mice (Figure 1). However, even mice of that treatment group later gained weight through subsequent treatments and returned to normal within one week (Figure 1).

Thirty-six wild-type B6 male and female mice (3 of each sex per treatment) were exposed by oropharyngeal aspiration to saline (control) or 1/10 SAF of oil and/or dispersant as described above on days 0, 7 and 13 before euthanasia on day 14. The left lung of each mouse was processed for total RNA sequencing and the right lung was processed for histology. Total RNA yield per mouse varied according to sex and treatment (Figure S2). As observed in the preliminary experiments, microscopic examination of tissue sections from the treated mice revealed areas of focal inflammation that were most prevalent in mice treated with either 1/10 SAF of Corexit 9500 or Corexit 9527. Through GAGE analysis [24] of total lung RNA prepared from the exposed mice, we identified three treatments, Corexit 9527, Corexit 9500 and 9527+oil, that induced significant differential expression at the KEGG pathway level (FDR < 0.10) (Table 1). The strongest effects were observed for Corexit 9527, as the treatment induced a large number of KEGG pathways, with 19 upregulated (Table 1, group 1) and four downregulated (Table 1, group 2). For the other two treatments, Corexit 9500 caused seven pathways upregulated (Table 1, group 3) and 9527+oil led to two pathways downregulated (Table 1, group 4).

**Figure 2:**
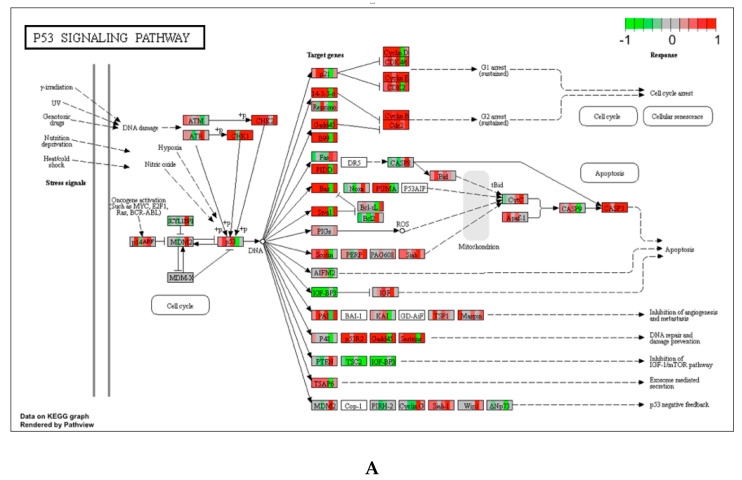

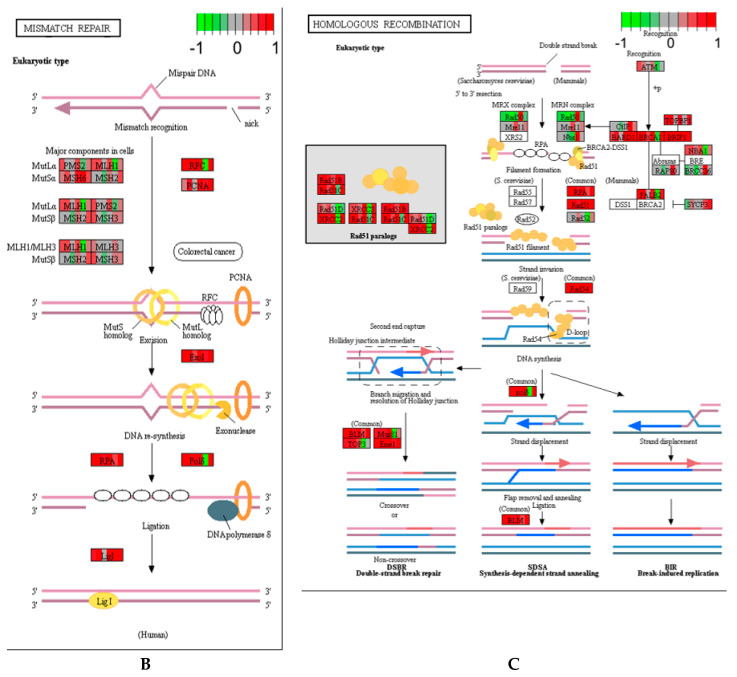

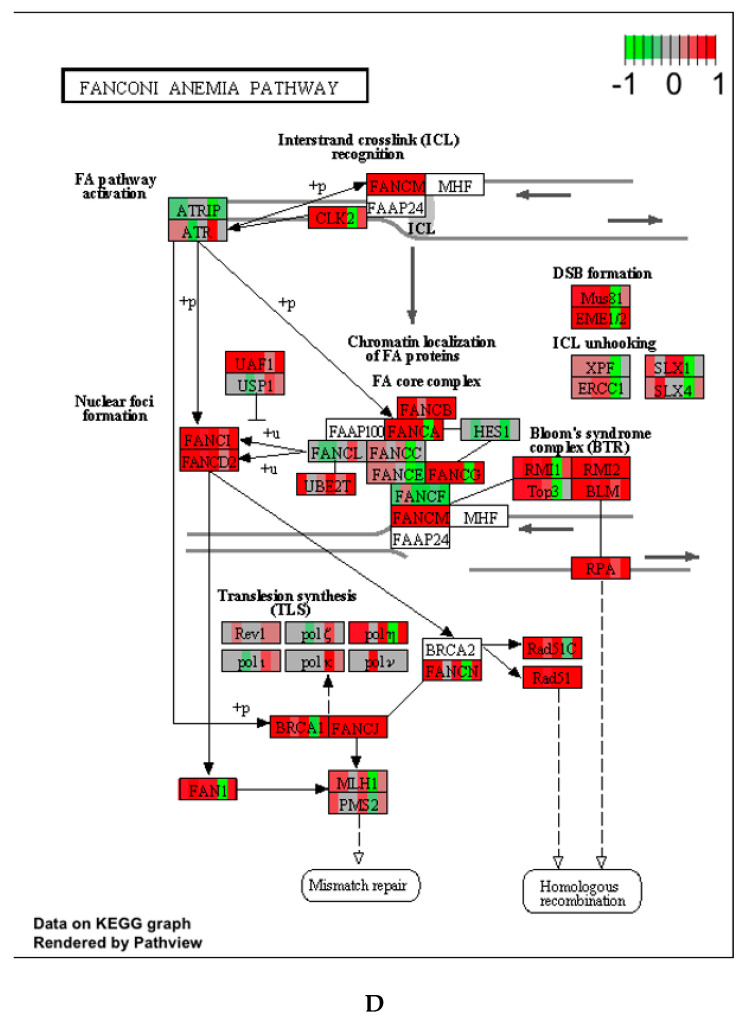
Example KEGG pathways upregulated by 1/10 SAF Corexit 9527. (**A**) p53 signaling pathway; (**B**) Mismatch repair pathway; (**C**) Homologous recombination pathway; (**D**) Fanconi anemia pathway. Red and green colors indicate up or downregulation, respectively, of gene expression for the mice in a treatment group.

Of note on the pathways upregulated by Corexit 9527, ~10 were related to responses to DNA damage (Table 1, group 1), as exemplified by p53 signaling, Fanconi anemia and mismatch repair. Most of the pathways related to DNA damage were also upregulated by Corexit 9500 (Table 1, group 3). Shown in Figure 2 are KEGG pathway plots for some example pathways related to DNA damage response, which were upregulated by Corexit 9527, including p53 signaling, mismatch repair, homologous recombination and Fanconi anemia pathways. The KEGG pathway plots were generated with the R package PathView [28]. As shown in the pathway plots, red colors are pervasive, suggesting an overall “turned-on”, i.e., upregulation, of the genes in the pathways.

To confirm the above results for upregulated pathways by Corexit 9527 related to DNA damage and repair, we submitted the upregulated genes (*p* < 0.05) to the online gene set enrichment analysis portal DAVID [25]. For Corexit 9527 treatment, several functional terms such as DNA damage, DNA repair, and cellular response to DNA damage stimulus all achieved significant enrichment in both male and female mice (Table 2).

We selected 11 of the most prominently activated genes related to DNA damage response and DNA repair for real-time PCR confirmation among mice treated with Corexit 9527 as compared to saline (as controls) (Table 3). All of the expressed genes from Corexit 9527 treated mice were confirmed using regression analysis of delta CT values (adjusting for sex effect) with significant *p* values (*p* < 0.05). Table 3 also shows the major functions of each listed gene related to DNA damage and repair.

### 3.3. Sex-Specific Analyses Identified Stronger Effects in Male Mice and Proinflammatory Pathways Upregulated by Oil in Male Mice

**Figure 3:**
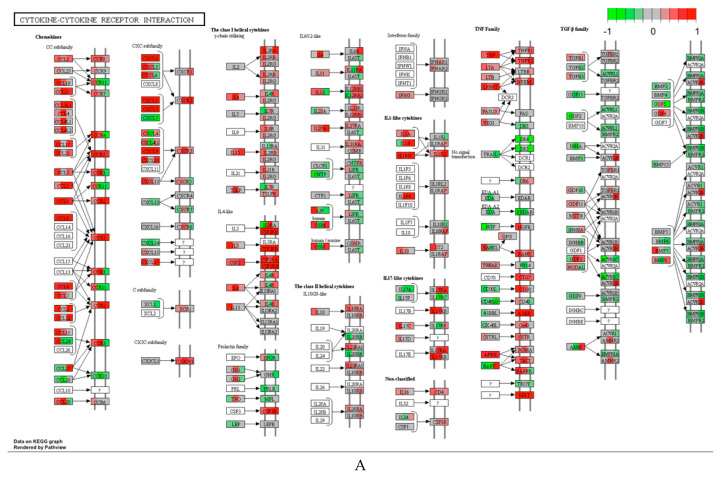

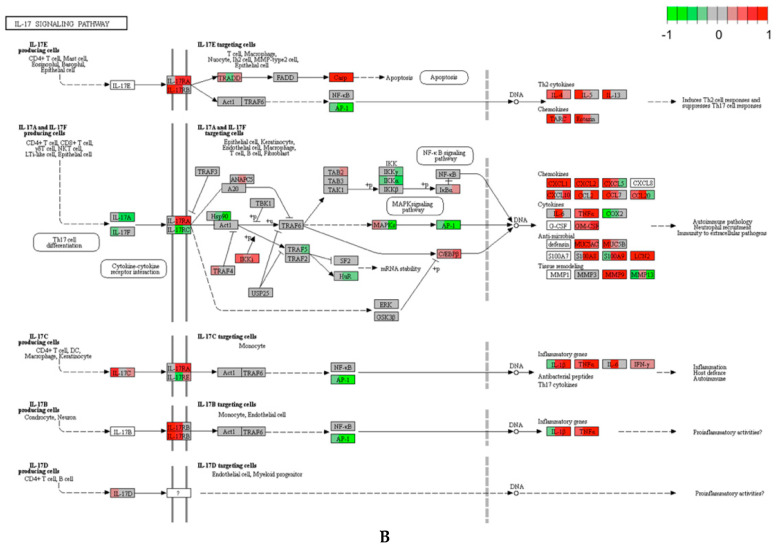
Example KEGG pathways upregulated by 1/10 SAF oil in male mice. (**A**) Cytokine-cytokine receptor interaction pathway; (**B**) IL-17 signaling pathway.

Differential expression analyses for each treatment was also performed in male and female mice, respectively. In general, treatment effects were stronger in male than female mice as more pathways were significantly altered relative to the saline control (FDR < 0.10) in male than female mice (Table 4, groups 1, 3, 4, 6 vs. groups 2, 5, 7, 8). For Corexit 9527, the major signatures were related to DNA damage responses, which were observed to be upregulated in both male and female mice, although the signals were stronger with more pathways upregulated in male than in female mice (Table 4, groups 1 and 2). Treatment with the MC252 crude oil SAF showed significant effects on male mice. Upregulation of proinflammatory pathways, such as cytokine–cytokine receptor interaction, IL-17 signaling, chemokine signaling and TNF signaling pathways were predominant (Table 4, group 4). As examples, Figure 3 shows two KEGG proinflammatory pathways, cytokine–cytokine receptor interaction and IL-17 signaling, which are upregulated by oil in male mice. The above upregulated pathways were not detected in the female mice treated with 1/10 oil SAF. As an exception, cytokine–cytokine receptor interaction pathway was observed downregulated in female mice treated with oil (Table 4, group 5).

Three pathways related to DNA damage response, cell cycle, DNA replication and homologous recombination were also upregulated in Corexit 9500-treated male mice (Table 4, group 6). Treatment of 9527+oil in female mice activated pathways related to tissue regeneration, such as Hippo, Notch, MAPK and Wnt signaling (Table 4, group 7).

To confirm the upregulated pathways related to DNA damage and repair by Corexit 9527 and the proinflammatory pathways upregulated by oil SAF treatment in male mice, we submitted to DAVID [25] the upregulated genes. Terms significantly enriched in the submitted genes from exposed male mice included innate immune response, chemokine-mediated signaling pathway, positive regulation of inflammatory response and cytokines (Table 2).

To confirm if there was downregulation of proinflammatory activities in female mice treated with oil SAF, the DAVID analysis of genes downregulated in female mice was performed and did not detect significant enrichment of any term related to inflammation, immune response and cytokine activities, etc.

The number of differentially expressed genes (*p* < 0.05) in pooled and sex-specific samples are shown in Supplemental Table S2.

### 3.4. Tumor-Bearing K-Ras^LA1^ Mice Showed Accelerated Tumorigenesis Induced by Oil and Dispersants

Eight week old tumor-bearing K-Ras^LA1^ (C57BL/6 background) mice were treated by oropharyngeal aspiration with 1/10 SAFs of oil and/or dispersants on days 0, 7 and 14 followed by sacrifice on day 21. For control purposes, K-Ras^LA1^ mice were treated with an equal volume of HBSS. The right lung of each mouse was fixed for analyses of lung tumor growth. Supplemental Figure S4 shows the box plots for the K-Ras^LA1^ mice tumor indices (pleural surface tumor count, tumor cell number and tumor burden). Supplemental Figure S5 shows an example of tumor nodules on the pleural surface of a K-RasLA-1 mice.

**Figure 4:**
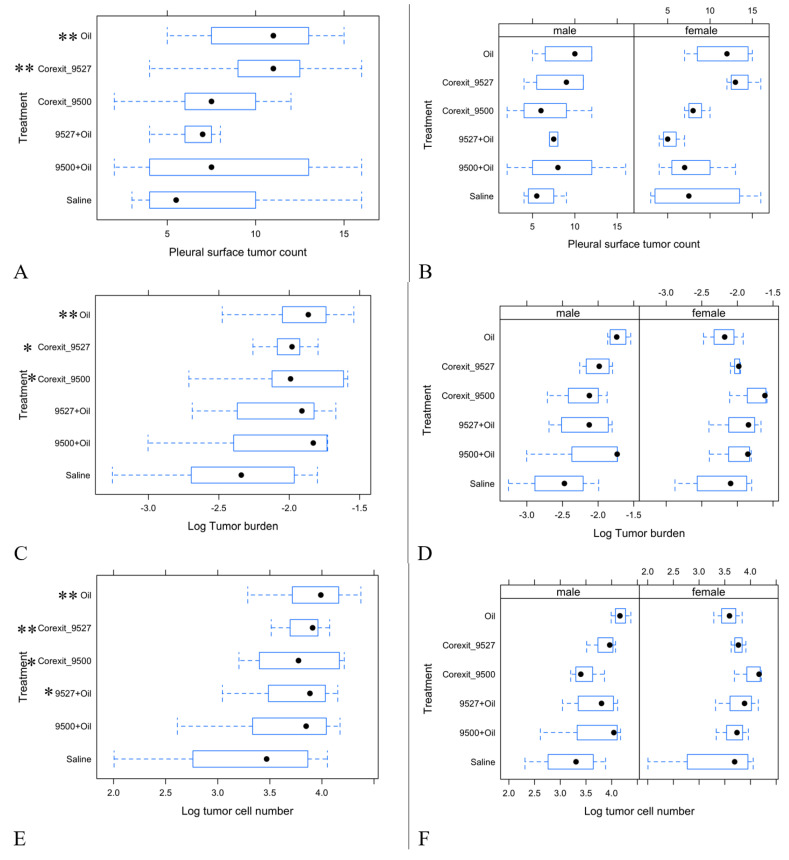
Box plots of K-Ras^LA1^ mice lung tumor indices across different treatments. (**A**) Box plot for the pleural surface tumor count for each treatment with combined sexes; (**B**) Box plot for pleural surface tumor count for each treatment and sex; (**C**) Box plot for tumor burden (log transformed) for each treatment for combined sexes; (**D**) Box plot for tumor burden (log transformed) for each treatment and sex; (**E**) Box plot for tumor cell number (log transformed) for each treatment for combined sexes; (**F**) Box plot for tumor cell number (log transformed) for each treatment and sex. Additionally shown in A, C and E are those groups whose lung tumor indices are significantly (*p* < 0.05, indicated by **) or marginally significantly (*p* < 0.10, indicated by *) elevated compared with saline control.

Comparison of tumor indices between a treatment group with control (Figure 4) suggested an increased pleural surface tumor count, tumor burden and tumor cell number in oil or Corexit 9527 treatments. Such an increase is more pronounced in the male group.

Based on regression analyses of association of each tumor index with different treatments, while adjusting for sex, oil treatment achieved a significant increase of pleural surface tumor count (*p* = 0.036), tumor burden (*p* = 0.030) and tumor cell number (*p* = 0.020) as compared with the control. Corexit 9527 caused significant increases of pleural surface tumor count (*p* = 0.026), and tumor cell number (*p* = 0.042) and a marginally significant increase of tumor burden (*p* = 0.073) as compared with the control. Corexit 9500 achieved a marginally significant increase of tumor burden (*p* = 0.087) and tumor cell number (*p* = 0.089) as compared with control. In addition, 9527+oil achieved a marginally significant increase of tumor cell number (*p* = 0.092) as compared with control.

Supplemental Figure S6 shows example images (one example subject from each treatment) of tumor burden characterization of K-Ras^LA-1^ mice under different treatments.

### 3.5. Upregulation of Cell Division/Cell Cycle Activities Promoted by Corexit 9527 is the Shared Signals with Previous RNA-seq Studies on Airway Epithelial Cells (the BEAS-2B Cell Line)

We compared the results of the current study (Table 1) with previous RNA-seq studies [13,14] on airway epithelial cells (the BEAS-2B cell line). The comparisons were performed at the KEGG pathway level and at the individual gene level and the similarities are shown in Table 5 and Table 6, respectively. At the pathway level, the consensus between the current study and the previous study [14] was achieved only for the Corexit 9527 treatment, where seven KEGG pathways were observed upregulated in both mouse lungs and BEAS-2B cells at a significance level of FDR < 0.10 (Table 5). The cell cycle pathway achieves the most significant meta-analysis *p* value (*p*= 4.22 × 10^−20^), suggesting the highest overall significance when combining the findings from two studies on the shared pathways. At the individual gene level, again, Corexit 9527 has the highest number of regulated genes (29 upregulated and 10 downregulated genes) shared between the current study and the previous study [13] (Table 6). Using DAVID enrichment analysis to annotate the 29 shared upregulated genes due to Corexit 9527 treatment (by submitting the 29 ENSG IDs), several significant functional terms, including Cell Division (FDR = 3.51 × 10^−8^), Mitosis (FDR = 1.22 × 10^−6^) and Cell Cycle (FDR = 5.25 × 10^−6^), were identified. In summary, the major common signal detected in both human airway epithelial cells [13,14] and mouse lung tissue is the upregulation of cell division and cell cycle activities promoted by Corexit 9527.

## 4. Discussion

This study builds upon previous reports [13,14] showing responses of human airway epithelial cells treated with fractions of oil and/or dispersant chemicals. In particular, Corexit 9527 treatment produced upregulated KEGG pathways related to carcinogenesis [14]. However, these findings were achieved through an in vitro system and hence their applicability to exposed humans needed to be investigated with an animal model.

Overall, the major signals detected in this study include upregulation of DNA damage/repair activities by Corexit 9527 treatment and enhanced proinflammatory activities by oil SAF treatment. Our results related to DNA damage due to Corexit 9527 and 9500 provide evidence to support the chemicals’ lung tumor promoting effects. DNA damage is a major mechanism underlying development of human cancers as damaged DNA may be replicated before repair, which gives rise to somatic mutations and altered proteins, resulting in cancer promotion [40]. Notably, in our study, in addition to DNA damage-related pathways, cell cycle and DNA replication pathways were also upregulated by Corexit 9527 and 9500 (Table 1 and Table 2). The increased cell cycle and DNA replication activities, a mechanism to replace damaged cells, may further replicate the somatic mutations generated from DNA damage initiated by dispersants, which may further enhance tumorigenesis.

Our results regarding proinflammatory signals in male mice due to oil SAF treatment also suggest contributions to lung carcinogenesis. An inflammatory microenvironment is one of the “hallmarks of cancer” [41]. Epidemiological evidence supports an important role of inflammation in lung cancer development [42]. A recent study comparing 807 incident lung cancer cases and 807 smoking-matched controls from three prospective cohorts identified a higher lung cancer risk for those participants with elevated concentrations of inflammatory cytokines, IL-6 and IL-8, suggesting an important role of inflammation in lung cancer etiology [43]. A similar association with prospective risk of lung cancer was achieved for other inflammatory markers, such as C-reactive protein, serum amyloid A and soluble tumor necrosis factor receptor 2 [44]. Moreover, we and others showed previously that interleukin-17 promotes lung tumor growth in murine models [45,46,47].

Using lung tumor bearing K-Ras^LA1^ mice, we tested the effects on tumorigenesis using the same treatments as in our RNA-seq studies. Interestingly, lung tumorigenesis indices of K-Ras^LA1^ mice were generally consistent with RNA-seq results of wild-type B6 mice in terms of signal intensities of significant pathways, such as those related to DNA damage, cell division and proinflammatory activities. The treatments that promoted lung tumorigenesis in K-Ras^LA1^ mice are oil and Corexit 9527, as might have been predicted from the RNA-seq results of B6 mice. Both of these two treatments led to significantly increased (*p* < 0.05) pleural surface tumors and tumor cell number in the lung. The two treatments also caused significant (*p* < 0.05), or marginally significant (*p* < 0.10), increases in tumor burden. In addition, Corexit 9500 treatment marginally increased (*p* < 0.10) both tumor burden and tumor cell number. This is also consistent with the intensity of signals of Corexit 9500 in the RNA-seq results.

Sex specificity is also observed in K-Ras^LA1^ mice, as in the wild-type B6 mice. As shown in Figure 4 (right panels), for the 1/10 SAF treatments of oil and Corexit 9527, the increase of tumorigenesis measures as compared to control (saline) were stronger in males than in females. This result is in agreement with the stronger signals in male than female mice for the RNA-seq results (Table 4). Overall, our K-Ras^LA1^ data provided consistent support for the expectations based on our RNA-seq data, where we predict that Corexit 9527 and oil treatments promote tumorigenesis due to the DNA damage and proinflammatory effects, respectively.

Similar to the sex-specific findings from our study, a recent study on the acute neurological effects of oil-spill cleanup-related exposures based on the Deepwater Horizon Oil Spill Coast Guard Cohort also reported sex-related neurological symptoms that were stronger in males than females [48]. We speculate that the sex difference in response to oil spill chemicals may be largely immune mediated as sex-specific differences in the immune response are well documented in the literature [49]. For example, peripheral blood mononuclear cells (PBMCs) from human males produce more TNF than PBMCs from females following lipopolysaccharide stimulation [50,51]. However, the sex difference in response to oil spill chemicals at the levels of gene expression and tumorigenesis in our study still needs to be further validated. For example, further testing in castrated or ovariectomized animals could implicate hormonal effects in the sex selective responses.

Toxicities of dispersants Corexit 9500 and 9527, which include predator/prey recognition, enzyme activity changes, effects on egg hatchability, and the threshold for bradycardia have been documented for animals [4]. Respiratory irritation symptoms associated with exposure to Corexit 9500 and 9527 have been reported in a portion of ~30,000 participants involved in Deepwater Horizon Oil Spill cleanup [6]. Similar symptoms, such as burning in the nose, throat and lungs, were also identified in women of southern Louisiana after the oil spill [7]. However, the long-term impacts of Corexit 9527 and 9500 and crude oil on the human respiratory system remain undetermined. Our previous RNA-seq-based studies with human airway epithelial cells [13,14] and the present study address public health concerns of Corexit 9500 and 9527 related to lung health.

The main difference in chemical components between the two types of Corexit is 2-butoxy ethanol, which is a major component of Corexit 9527 but not included in Corexit 9500 [4]. The presence of 2-butoxy ethanol may account for the higher toxicity of Corexit 9527 relative to Corexit 9500, as suggested in our RNA-seq data. However, the comparison of lethality and toxicity of the two kinds of Corexit is still inconclusive. As reviewed in [4], Corexit 9527 may be more lethal and toxic than Corexit 9500 to invertebrates but vice versa for fish. No comparison of the relative toxicities of the two dispersants has been tested for birds and mammals yet [4].

The overlap between the current study and our previous RNA-seq study on human airway epithelial cells [13,14] is limited (Table 5). This may have been expected beforehand due to the limitations of a single cell culture compared with lung tissue of greater cellular complexity. Indeed, the limitations of the previous studies [13,14] are the reasons we initiated the current study. Despite the differences, comparison of the current study with the previous ones [13,14] does provide some interesting results; the overlap is mainly observed for the effects of Corexit 9527. The main signal of Corexit 9527 treatment in both an airway epithelial cell line and lung tissue is the upregulation of cell cycle activities (as shown in both pathway level analysis and in enrichment analysis of overlapping genes), which once again provides support for the tumor promoting potential of Corexit 9527. In that regard, an increase of cell cycle activities is a common observation in carcinogenesis and cell cycle proteins are promising targets in cancer therapy [52].

## 5. Conclusions

Our study, for the first time, identified potential carcinogenic effects of Corexit 9527/9500 and crude oil to the lung system at the mouse gene expression level. Using a mutant K-ras-expressing mouse model, we also demonstrated for the first time accelerated tumorigenesis associated with exposure to crude oil or Corexit 9527/9500. Taken together, we obtained evidence supporting the potential respiratory harm of chronic exposure to oil and dispersants for the general population, especially people working in the oil industry. The next phase of the study may involve genotoxic and immunologic analyses, such as identification of lung somatic mutations that may arise after long-term exposure and characterization of lung immune cells of exposed mice.

## Figures and Tables

**Table 1:** Significantly regulated pathways by different treatments.

Group	Differentially Regulated Pathways	*p* Value	FDR	Treatment	Direction of Regulation
1	mmu04110 Cell cycle	1.03 × 10^−16^	2.33 × 10^−14^	Corexit 9527	Upregulation
	mmu03030 DNA replication	3.52 × 10^−13^	3.98 × 10^−11^		
	mmu03440 Homologous recombination	3.89 × 10^−9^	2.93 × 10^−7^		
	mmu03460 Fanconi anemia pathway	2.83 × 10^−7^	1.60 × 10^−5^		
	mmu04115 p53 signaling pathway	3.06 × 10^−6^	1.16 × 10^−4^		
	mmu03430 Mismatch repair	3.08 × 10^−6^	1.16 × 10^−4^		
	mmu04142 Lysosome	1.39 × 10^−4^	4.48 × 10^−3^		
	mmu03420 Nucleotide excision repair	2.44 × 10^−4^	6.91 × 10^−3^		
	mmu03410 Base excision repair	2.88 × 10^−4^	7.24 × 10^−3^		
	mmu04145 Phagosome	7.42 × 10^−4^	1.56 × 10^−2^		
	mmu03013 RNA transport	7.59 × 10^−4^	1.56 × 10^−2^		
	mmu00520 Amino sugar and nucleotide sugar metabolism	2.35 × 10^−3^	4.42 × 10^−2^		
	mmu00670 One carbon pool by folate	2.71 × 10^−3^	4.44 × 10^−2^		
	mmu04914 Progesterone-mediated oocyte maturation	2.75 × 10^−3^	4.44 × 10^−2^		
	mmu04114 Oocyte meiosis	3.67 × 10^−3^	5.53 × 10^−2^		
	mmu00051 Fructose and mannose metabolism	4.16 × 10^−3^	5.57 × 10^−2^		
	mmu04610 Complement and coagulation cascades	4.33 × 10^−3^	5.57 × 10^−2^		
	mmu00240 Pyrimidine metabolism	4.44 × 10^−3^	5.57 × 10^−2^		
	mmu03040 Spliceosome	7.76 × 10^−3^	9.23 × 10^−2^		
2	mmu04022 cGMP-PKG signaling pathway	6.46 × 10^−4^	7.42 × 10^−2^	Corexit 9527	Downregulation
	mmu04015 Rap1 signaling pathway	7.63 × 10^−4^	7.42 × 10^−2^		
	mmu04925 Aldosterone synthesis and secretion	1.01 × 10^−3^	7.42 × 10^−2^		
	mmu04261 Adrenergic signaling in cardiomyocytes	1.31 × 10^−3^	7.42 × 10^−2^		
3	mmu04110 Cell cycle	5.15 × 10^−8^	1.16 × 10^−5^	Corexit 9500	Upregulation
	mmu03030 DNA replication	3.57 × 10^−7^	4.04 × 10^−5^		
	mmu04142 Lysosome	1.69 × 10^−4^	1.27 × 10^−2^		
	mmu03410 Base excision repair	9.16 × 10^−4^	4.86 × 10^−2^		
	mmu04115 p53 signaling pathway	1.11 × 10^−3^	4.86 × 10^−2^		
	mmu03440 Homologous recombination	1.29 × 10^−3^	4.86 × 10^−2^		
	mmu03430 Mismatch repair	2.78 × 10^−3^	8.99 × 10^−2^		
4	mmu00190 Oxidative phosphorylation	2.56 × 10^−4^	5.79 × 10^−2^	9527+oil	Downregulation
	mmu03008 Ribosome biogenesis in eukaryotes	6.40 × 10^−4^	7.23 × 10^−2^		

**Table 2:** DAVID gene set enrichment analysis of genes upregulated by Corexit 9527 or oil.

Corexit 9527 (All Mice)			
Category	Term	Fold Enrichment	Bonferroni Corrected *p* Value
UP_KEYWORDS	DNA damage	4.05	9.30 × 10^−11^
UP_KEYWORDS	DNA repair	4.39	1.63 × 10^-10^
GOTERM_BP_DIRECT	GO:0006974~cellular response to DNA damage stimulus	3.22	7.30 × 10^−8^
GOTERM_BP_DIRECT	GO:0006281~DNA repair	3.57	1.91 × 10^−7^
**Corexit 9527 (male mice)**			
**Category**	**Term**	**Fold Enrichment**	**Bonferroni corrected *p* value**
UP_KEYWORDS	DNA damage	2.72	2.34 × 10^−8^
UP_KEYWORDS	DNA repair	2.85	9.77 × 10^−8^
GOTERM_BP_DIRECT	GO:0006974~cellular response to DNA damage stimulus	2.23	1.40 × 10^−5^
GOTERM_BP_DIRECT	GO:0006281~DNA repair	2.44	2.15 × 10^−5^
**Corexit 9527 (female mice)**			
**Category**	**Term**	**Fold Enrichment**	**Bonferroni corrected *p* value**
GOTERM_BP_DIRECT	GO:0006974~cellular response to DNA damage stimulus	5.57	0.012
UP_KEYWORDS	DNA damage	6.26	0.014
UP_KEYWORDS	DNA repair	6.70	0.028
**Oil (male mice)**			
**Category**	**Term**	**Fold Enrichment**	**Bonferroni corrected *p* value**
GOTERM_BP_DIRECT	GO:0045087~innate immune response	3.39	3.09 × 10^−12^
GOTERM_BP_DIRECT	GO:0070098~chemokine-mediated signaling pathway	7.65	2.05 × 10^−7^
GOTERM_BP_DIRECT	GO:0050729~positive regulation of inflammatory response	5.93	1.42 × 10^−4^
UP_KEYWORDS	Cytokine	3.22	3.44 × 10^−4^

**Table 3:** Realtime PCR results.

Gene	Function of Gene	Reference	Fold Change	*p* Value
Chek1	checkpoint mediated cell cycle arrest in response to DNA damage	[29]	2.20	7.6 × 10^−4^
Rad51ap1	participate in a common DNA damage response pathway associated with the activation of homologous recombination and double-strand break repair	[30]	2.35	1.5 × 10^−4^
DTL	cell cycle control, DNA damage response and translesion DNA synthesis	[31]	2.54	3.12 × 10^−4^
Clspn	checkpoint arrest of the cell cycle in response to replicative stress or DNA damage	[32]	3.23	5.39 × 10^−3^
Fen1	Structure-specific nuclease with 5′-flap endonuclease and 5′-3′ exonuclease activities involved in DNA replication and repair.	[33]	1.29	3.46 × 10^−2^
PCLAF	PCNA-binding protein that acts as a regulator of DNA repair during DNA replication	[34]	2.56	1.43 × 10^−4^
Ticrr	involved in the initiation of DNA replication	[35]	1.91	1.19 × 10^−2^
Uhrf1	functions in the p53-dependent DNA damage checkpoint	[36]	2.64	2.83 × 10^−4^
Chaf1b	mediates chromatin assembly in DNA replication and DNA repair	[37]	1.82	7.23 × 10^−4^
Bard1	Plays a central role in the control of the cell cycle in response to DNA damage	[38]	1.37	1.28 × 10^−2^
Chek2	is activated when DNA becomes damaged or when DNA strands break	[39]	1.26	9.83 × 10^−4^

Note: For each gene, a statistically significant (*p* < 0.05) negative regression coefficient was achieved for the treatment Corexit 9527 in regression analysis, suggesting a significant lower CT value (and hence a higher expression level) for the treatment as compared with the control treatment. The fold change is for the expression in Corexit 9527 vs. control treatment (saline).

**Table 4:** Sex-specific analysis for regulation of KEGG pathways.

Group	Differentially Regulated Pathways	*p* Value	FDR	Treatment	Direction of Regulation	Sex
1	mmu04110 Cell cycle	3.41 × 10^−15^	7.60 × 10^−16^	Corexit 9527	Upregulation	Male
	mmu03030 DNA replication	4.52 × 10^−11^	5.04 × 10^−9^			
	mmu03440 Homologous recombination	3.31 × 10^−7^	2.46 × 10^−5^			
	mmu03460 Fanconi anemia pathway	2.31 × 10^−5^	1.09 × 10^−3^			
	mmu03010 Ribosome	2.45 × 10^−5^	1.09 × 10^−3^			
	mmu03430 Mismatch repair	5.36 × 10^−5^	1.72 × 10^−3^			
	mmu04115 p53 signaling pathway	5.41 × 10^−5^	1.72 × 10^−3^			
	mmu03410 Base excision repair	4.55 × 10^−4^	1.14 × 10^−2^			
	mmu04142 Lysosome	4.61 × 10^−4^	1.14 × 10^−2^			
	mmu04610 Complement and coagulation cascades	1.19 × 10^−3^	2.64 × 10^−2^			
	mmu03420 Nucleotide excision repair	1.30 × 10^−3^	2.64 × 10^−2^			
	mmu04145 Phagosome	2.03 × 10^−3^	3.78 × 10^−2^			
	mmu03040 Spliceosome	2.79 × 10^−3^	4.79 × 10^−2^			
	mmu04060 Cytokine−cytokine receptor interaction	3.41 × 10^−3^	5.35 × 10^−2^			
	mmu03013 RNA transport	3.73 × 10^−3^	5.35 × 10^−2^			
	mmu00240 Pyrimidine metabolism	3.92 × 10^−3^	5.35 × 10^−2^			
	mmu00051 Fructose and mannose metabolism	4.08 × 10^−3^	5.35 × 10^−2^			
	mmu00520 Amino sugar and nucleotide sugar metabolism	5.40 × 10^−3^	6.69 × 10^−2^			
	mmu04657 IL-17 signaling pathway	5.78 × 10^−3^	6.79 × 10^−2^			
	mmu03050 Proteasome	6.39 × 10^−3^	7.13 × 10^−2^			
	mmu00052 Galactose metabolism	7.73 × 10^−3^	8.21 × 10^−2^			
2	mmu04110 Cell cycle	5.03 × 10^−6^	1.12 × 10^−3^	Corexit 9527	Upregulation	Female
	mmu03030 DNA replication	1.37 × 10^−5^	1.52 × 10^−3^			
	mmu03440 Homologous recombination	2.60 × 10^−4^	1.93 × 10^−2^			
	mmu03460 Fanconi anemia pathway	6.39 × 10^−4^	3.55 × 10^−2^			
	mmu03430 Mismatch repair	2.10 × 10^−3^	9.32 × 10^−2^			
3	mmu04925 Aldosterone synthesis and secretion	1.86 × 10^−4^	2.09 × 10^−2^	Corexit 9527	Downregulation	Male
	mmu04923 Regulation of lipolysis in adipocytes	2.15 × 10^−4^	2.09 × 10^−2^			
	mmu04022 cGMP-PKG signaling pathway	2.81 × 10^−4^	2.09 × 10^−2^			
	mmu04015 Rap1 signaling pathway	3.84 × 10^−4^	2.14 × 10^−2^			
	mmu04024 cAMP signaling pathway	5.26 × 10^−4^	2.35 × 10^−2^			
	mmu04261 Adrenergic signaling in cardiomyocytes	7.49 × 10^−4^	2.53 × 10^−2^			
	mmu04723 Retrograde endocannabinoid signaling	7.96 × 10^−4^	2.53 × 10^−2^			
	mmu04728 Dopaminergic synapse	1.47 × 10^−3^	4.11 × 10^−2^			
	mmu04972 Pancreatic secretion	2.87 × 10^−3^	7.12 × 10^−2^			
	mmu04270 Vascular smooth muscle contraction	3.24 × 10^−3^	7.22 × 10^−2^			
	mmu04713 Circadian entrainment	4.68 × 10^−3^	9.50 × 10^−2^			
	mmu04916 Melanogenesis	5.36 × 10^−3^	9.97 × 10^−2^			
4	mmu04060 Cytokine-cytokine receptor interaction	6.49 × 10^−10^	1.45 × 10^−7^	Oil	Upregulation	Male
	mmu04145 Phagosome	6.92 × 10^−6^	5.07 × 10^−4^			
	mmu04657 IL-17 signaling pathway	7.66 × 10^−6^	5.07 × 10^−4^			
	mmu04062 Chemokine signaling pathway	9.09 × 10^−6^	5.07 × 10^−4^			
	mmu04110 Cell cycle	3.35 × 10^−5^	1.49 × 10^−3^			
	mmu04621 NOD-like receptor signaling pathway	6.56 × 10^−5^	2.41 × 10^−3^			
	mmu04640 Hematopoietic cell lineage	7.57 × 10^−5^	2.41 × 10^−3^			
	mu04142 Lysosome	1.13 × 10^−4^	3.16 × 10^−3^			
	mmu0462 Toll−like receptor signaling pathway	2.57 × 10^−4^	6.37 × 10^−3^			
	mmu03030 DNA replication	3.25 × 10^−4^	7.25 × 10^−3^			
	mmu04610 Complement and coagulation cascades	6.09 × 10^−4^	1.13 × 10^−2^			
	mmu04668 TNF signaling pathway	6.10 × 10^−4^	1.13 × 10^−2^			
	mmu04380 Osteoclast differentiation	1.18 × 10^−3^	2.02 × 10^−2^			
	mmu04666 Fc gamma R-mediated phagocytosis	1.31 × 10^−3^	2.08 × 10^−2^			
	mmu03440 Homologous recombination	2.31 × 10^−3^	3.35 × 10^−2^			
	mmu04664 Fc epsilon RI signaling pathway	2.44 × 10^−3^	3.35 × 10^−2^			
	mmu04672 Intestinal immune network for IgA production	2.55 × 10^−3^	3.35 × 10^−2^			
	mmu04623 Cytosolic DNA-sensing pathway	4.47 × 10^−3^	5.53 × 10^−2^			
5	mmu04060 Cytokine-cytokine receptor interaction	2.03 × 10^−4^	4.50 × 10^−2^	Oil	Downregulation	Female
6	mmu04110 Cell cycle	2.22 × 10^−7^	4.95 × 10^−5^	Corexi 9500	Upregulation	Male
	mmu03030 DNA replication	8.24 × 10^−6^	9.19 × 10^−4^			
	mmu03440 Homologous recombination	3.72 × 10^−4^	2.77 × 10^−2^			
7	mmu04390 Hippo signaling pathway	3.15 × 10^−5^	6.70 × 10^−3^	9527+oil	Upregulation	Female
	mmu04330 Notch signaling pathway	6.03 × 10^−5^	6.70 × 10^−3^			
	mmu04360 Axon guidance	1.85 × 10^−4^	1.37 × 10^−2^			
	mmu04510 Focal adhesion	3.60 × 10^−4^	2.00 × 10^−2^			
	mmu04010 MAPK signaling pathway	1.10 × 10^−3^	4.90 × 10^−2^			
	mmu04310 Wnt signaling pathway	1.75 × 10^−3^	6.46 × 10^−2^			
	mmu04810 Regulation of actin cytoskeleton	2.29 × 10^−3^	7.26 × 10^−2^			
8	mmu00190 Oxidative phosphorylation	5.88 × 10^−5^	1.20 × 10^−2^	9527+oil	Downregulation	Female
	mmu03010 Ribosome	1.08 × 10^−4^	1.20 × 10^−2^			
	mmu04060 Cytokine-cytokine receptor interaction	8.68 × 10^−4^	6.43 × 10^−2^			

**Table 5:** Comparison between this study and RNA-seq analyses of a treated human airway epithelial cell line (BEAS-2B) [14] at pathway level.

Differentially Regulated Pathways	BEAS-2B Cell [13]		Mice		Meta-Analysis *p* Value	Treatment	Direction of Regulation
	*p* Value	FDR	*p* Value	FDR			
Cell cycle	4.10 × 10^−4^	5.53 × 10^−3^	1.03 × 10^−16^	2.33 × 10^−14^	4.22 × 10^−20^	Corexit9527	Upregulation
RNA transport	2.03 × 10^−9^	1.64 × 10^−7^	7.59 × 10^−4^	1.56 × 10^−2^	1.54 × 10^−12^		
Spliceosome	6.09 × 10^−7^	1.97 × 10^−5^	7.76 × 10^−3^	9.23 × 10^−2^	4.73 × 10^−9^		
Pyrimidine metabolism	6.59 × 10^−5^	1.53 × 10^−3^	4.44 × 10^−3^	5.57 × 10^−2^	2.93 × 10^−7^		
Oocyte meiosis	6.65 × 10^−4^	8.29 × 10^−3^	3.67 × 10^−3^	5.53 × 10^−2^	2.44 × 10^−6^		
Amino sugar and nucleotide sugar metabolism	6.48 × 10^−3^	5.77 × 10^−2^	2.35 × 10^−3^	4.42 × 10^−2^	1.52 × 10^−5^		
Progesterone-mediated oocyte maturation	8.47 × 10^−3^	6.54 × 10^−2^	2.75 × 10^−3^	4.44 × 10^−2^	2.33 × 10^−5^		

**Table 6:** Comparison between this study and RNA-seq analyses of a treated human airway epithelial cell line (BEAS-2B) [13] at gene level.

Differentially Regulated Genes	BEAS-2B Cell [14]		Mice		Direction of Regulation	Treatment
	Ensemb ID	*p* Value	EnsembI ID	*p* Value		
TRIM59	ENSG00000213186	5.98 × 10^−3^	ENSMUSG00000034317	3.40 × 10^−2^	Upregulation	Oil
RAB11FIP1	ENSG00000156675	1.10 × 10^−2^	ENSMUSG00000031488	2.53 × 10^−2^	Downregulation	
EGR2	ENSG00000122877	2.78 × 10^−2^	ENSMUSG00000037868	2.34 × 10^−2^	Upregulation	Corexit 9500
ITGA11	ENSG00000137809	4.19×10^−2^	ENSMUSG00000032243	4.25 × 10^−2^		
ETV4	ENSG00000175832	3.90 × 10^−2^	ENSMUSG00000017724	4.18 × 10^−3^		
KCNG1	ENSG00000026559	2.04 × 10^−2^	ENSMUSG00000074575	1.96 × 10^−2^		
ATXN7L1	ENSG00000146776	2.28 × 10^−2^	ENSMUSG00000020564	2.11 × 10^−2^		
DCP1B	ENSG00000151065	1.15 × 10^−2^	ENSMUSG00000041477	4.49 × 10^−2^		
TIMP1	ENSG00000102265	3.25 × 10^−2^	ENSMUSG00000001131	3.40 × 10^−2^		
DLC1	ENSG00000164741	3.43 × 10^−2^	ENSMUSG00000031523	2.90 × 10^−2^	Downregulation	
STX2	ENSG00000111450	1.12 × 10^−2^	ENSMUSG00000029428	3.94 × 10^−3^		
MSI2	ENSG00000153944	3.58 × 10^−2^	ENSMUSG00000069769	3.50 × 10^−2^		
LATS2	ENSG00000150457	9.04 × 10^−3^	ENSMUSG00000021959	3.31 × 10^−2^		
ING2	ENSG00000168556	1.06 × 10^−2^	ENSMUSG00000063049	1.84 × 10^−2^		
MID1	ENSG00000101871	3.46 × 10^−2^	ENSMUSG00000035299	5.75 × 10^−3^		
RAB11FIP1	ENSG00000156675	4.13 × 10^−3^	ENSMUSG00000031488	3.42 × 10^−2^		
TGFBR1	ENSG00000106799	7.39 × 10^−4^	ENSMUSG00000021569	3.65 × 10^−2^		
ABI3BP	ENSG00000154175	3.02 × 10^−4^	ENSMUSG00000035258	4.16 × 10^−2^		
FAM198B	ENSG00000164125	7.29 × 10^−3^	ENSMUSG00000027955	3.51 × 10^−2^		
PNO1	ENSG00000115946	3.25 × 10^−2^	ENSMUSG00000020116	9.38 × 10^−3^	Upregulation	Corexit 9527
CCT5	ENSG00000150753	3.60 × 10^−2^	ENSMUSG00000022234	3.04 × 10^−2^		
NA	ENSG00000120254	4.07 × 10^−2^	ENSMUSG00000040675	4.96×10^−3^		
PLK1	ENSG00000166851	3.02×10^−2^	ENSMUSG00000030867	3.81 × 10^−7^		
HMMR	ENSG00000072571	4.07 × 10^−2^	ENSMUSG00000020330	8.67 × 10^−10^		
CPOX	ENSG00000080819	3.19 × 10^−2^	ENSMUSG00000022742	4.08 × 10^−2^		
SND1	ENSG00000197157	1.26 × 10^−2^	ENSMUSG00000001424	2.67 × 10^−5^		
PRR11	ENSG00000068489	7.37 × 10^−3^	ENSMUSG00000020493	3.77 × 10^−3^		
IMPDH2	ENSG00000178035	1.90 × 10^−2^	ENSMUSG00000062867	3.51 × 10^−3^		
MAD2L1	ENSG00000164109	2.11 × 10^−2^	ENSMUSG00000029910	2.27 × 10^−6^		
CCNA2	ENSG00000145386	1.64 × 10^−2^	ENSMUSG00000027715	6.64 × 10^−5^		
XRCC6	ENSG00000196419	3.52 × 10^−2^	ENSMUSG00000022471	2.91 × 10^−2^		
TRIP13	ENSG00000071539	4.51 × 10^−2^	-	1.76 × 10^−2^		
NA	ENSG00000143179	3.99 × 10^−2^	ENSMUSG00000026558	4.05 × 10^−3^		
CKS1B	ENSG00000173207	3.26 × 10^−2^	ENSMUSG00000028044	1.95 × 10^−2^		
MAD1L1	ENSG00000002822	2.09 × 10^−2^	ENSMUSG00000029554	4.80 × 10^−3^		
TPX2	ENSG00000088325	2.35 × 10^−2^	ENSMUSG00000027469	1.07 × 10^−3^		
HDGF	ENSG00000143321	1.63 × 10^−2^	ENSMUSG00000004897	6.56 × 10^−3^		
RACGAP1	ENSG00000161800	3.97 × 10^−2^	ENSMUSG00000023015	8.94 × 10^−9^		
ORC1	ENSG00000085840	1.34 × 10^−2^	ENSMUSG00000028587	6.08 × 10^−4^		
PBK	ENSG00000168078	4.67 × 10^−2^	ENSMUSG00000022033	1.29 × 10^−5^		
NCAPD2	ENSG00000010292	2.04 × 10^−3^	ENSMUSG00000038252	3.53 × 10^−2^		
NEK2	ENSG00000117650	4.64 × 10^−2^	ENSMUSG00000026622	3.81 × 10^−3^		
BIRC5	ENSG00000089685	4.71 × 10^−2^	ENSMUSG00000017716	4.84 × 10^−8^		
HDLBP	ENSG00000115677	2.24 × 10^−2^	ENSMUSG00000034088	4.82 × 10^−2^		
RRM1	ENSG00000167325	4.51 × 10^−2^	ENSMUSG00000030978	3.24 × 10^−7^		
CCNF	ENSG00000162063	2.36 × 10^−2^	ENSMUSG00000072082	7.81 × 10^−4^		
CEP85	ENSG00000130695	3.83 × 10^−2^	ENSMUSG00000037443	3.98 × 10^−3^		
GTSE1	ENSG00000075218	3.26 × 10^−2^	ENSMUSG00000022385	1.55 × 10^−4^		
SCYL3	ENSG00000000457	2.74 × 10^−2^	ENSMUSG00000026584	3.17×10^−2^	Downregulation	
TSNAXIP1	ENSG00000102904	4.61×10^−2^	ENSMUSG00000031893	9.47×10^−3^		
TDRP	ENSG00000180190	5.61×10^−3^	ENSMUSG00000050052	6.78 × 10^−3^		
SAPCD1	ENSG00000228727	3.33 × 10^−2^	ENSMUSG00000036185	3.72 × 10^−2^		
NFASC	ENSG00000163531	4.39 × 10^−2^	ENSMUSG00000026442	5.92 × 10^−4^		
LYPD5	ENSG00000159871	4.60 × 10^−2^	ENSMUSG00000030484	9.68 × 10^−3^		
MYH7B	ENSG00000078814	1.20 × 10^−2^	ENSMUSG00000074652	1.85 × 10^−2^		
SNCG	ENSG00000173267	1.14 × 10^−3^	ENSMUSG00000023064	1.82 × 10^−2^		
EGR1	ENSG00000120738	4.22 × 10^−2^	ENSMUSG00000038418	1.53 × 10^−2^		
FOS	ENSG00000170345	3.03 × 10^−2^	ENSMUSG00000021250	4.43 × 10^−2^		
VCL	ENSG00000035403	1.38 × 10^−2^	ENSMUSG00000021823	3.78 × 10^−2^	Upregulation	9500+oil
FSTL3	ENSG00000070404	4.14 × 10^−2^	ENSMUSG00000020325	4.84 × 10^−2^		
SOCS2	ENSG00000120833	3.96 × 10^−2^	ENSMUSG00000020027	1.05 × 10^−2^		
SERGEF	ENSG00000129158	3.89 × 10^−2^	ENSMUSG00000030839	1.29 × 10^−2^	Downregulation	
CCNG2	ENSG00000138764	1.13 × 10^−2^	ENSMUSG00000029385	3.62 × 10^−2^		
COL14A1	ENSG00000187955	4.39 × 10^−2^	ENSMUSG00000022371	4.92 × 10^−2^		
TMEM150C	ENSG00000249242	2.51 × 10^−2^	ENSMUSG00000050640	2.93 × 10^−2^	Upregulation	9527+oil
PRSS23	ENSG00000150687	5.18 × 10^−3^	ENSMUSG00000039405	3.30 × 10^−2^		
CSF3R	ENSG00000119535	4.22 × 10^−2^	ENSMUSG00000028859	1.90 × 10^−2^		
TRNT1	ENSG00000072756	4.64 × 10^−2^	ENSMUSG00000013736	4.79 × 10^−2^	Downregulation	
LRRC59	ENSG00000108829	4.02 × 10^−2^	ENSMUSG00000020869	3.38 × 10^−2^		
MRPL30	ENSG00000241962	2.50 × 10^−2^	ENSMUSG00000026087	3.32 × 10^−2^		
CLPB	ENSG00000162129	3.75 × 10^−2^	ENSMUSG00000001829	3.35 × 10^−2^		
NA	ENSG00000105793	2.66 × 10^−2^	ENSMUSG00000040464	3.50 × 10^−2^		
MTSS1	ENSG00000170873	1.19 × 10^−2^	ENSMUSG00000022353	2.98 × 10^−2^		

Note: 1. In table, the meta-analysis *p* value was achieved using Fisher’s method (20) to combine the *p* values in the two studies so as to summarize the overall significance of each pathway across the two studies. 2. For some ENSG Ensembl IDs in table, there are no respective HGNC (HUGO Gene Nomenclature Committee) symbols. Hence, their gene names in first column are shown as NA.

## Supplementary Materials

The following are available online at https://www.mdpi.com/1660-4601/17/15/5466/s1, Figure S1: Preparation of saline accommodated fraction (SAF). Figure S2: RNA yield from treated mice. Figure S3. Pathological changes associated with exposure of mice to oil and/or dispersant in preliminary experiments. Figure S4: Box plots for the K-Ras^LA1^ mice lung tumor indices. Figure S5: Representative tumor nodules on the pleural surface of a K-Ras^LA1^ mouse. Figure S6: Tumor histopathology of K-Ras^LA1^ mice. Table S1: Primers for qRT-PCR experiments. Table S2. Number of differentially expressed genes for different sexes and treatments.
