# Supplementary material for: The Impact of the Deepwater Horizon Oil Spill upon Lung Health—Mouse Model-Based RNA-Seq Analyses
Source: Int J Environ Res Public Health. 2020 Jul 29;17(15):5466. doi: 10.3390/ijerph17155466 (PMC7432840; doi:10.3390/ijerph17155466)
Supplement: Supplementary file 1 [file ijerph-17-05466-s001.pdf]

Supplemental Figure 1

A.

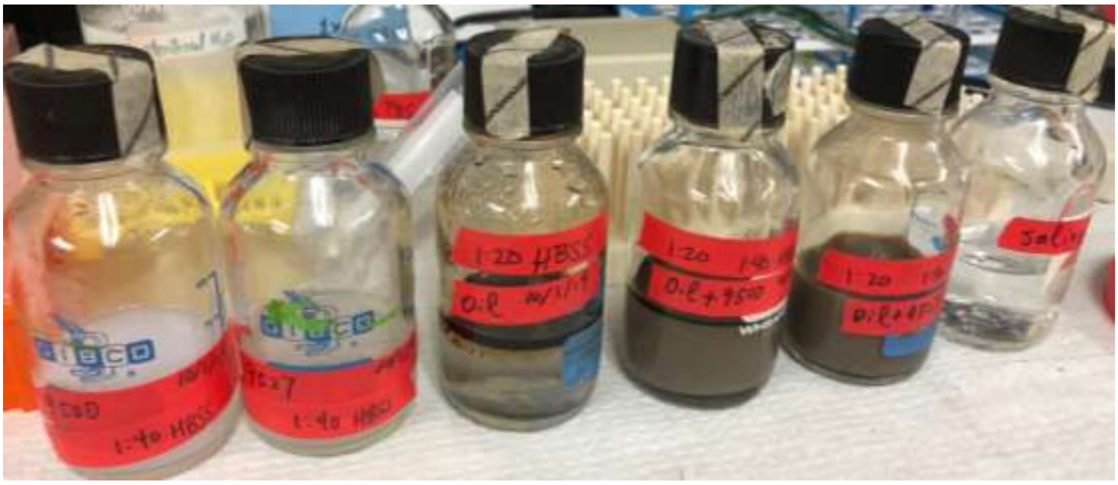

B.

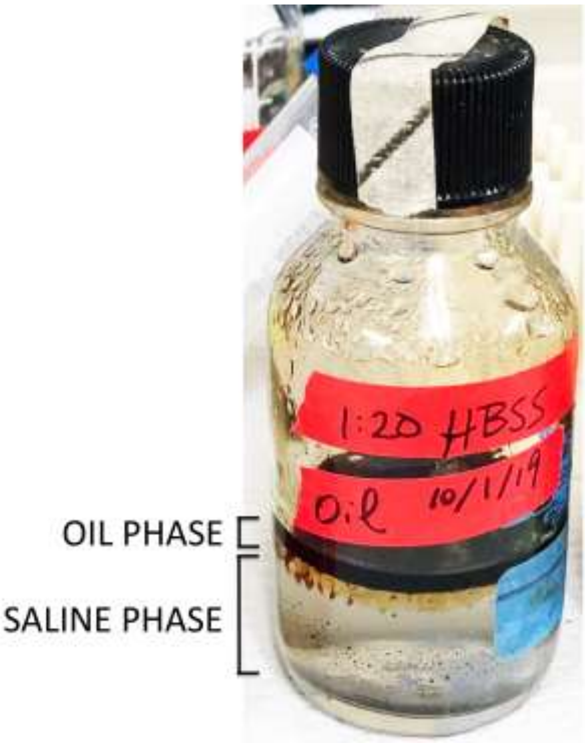

Supplemental Figure 1. Preparation of saline accommodated fraction (SAF). (A) MC252 oil, Corexit 9500, Corexit 9527, oil+9500 and oil +9527 were mixed and equilibrated in Hank's balanced salts solution at ratios of 1:20 (oil) and 1:40 (dispersants). Shown from left to right are SAF of Corexit 9500, Corexit 9527, MC252 oil, oil+9500, oil+9527 and saline (control). Photos of SAF were taken after letting the solutions stand for 1 hr after mixing. The oil alone sample has a prominent phase separation, and there was a small amount of phase separation for the 9500+oil sample. The other samples did not have phase separation. **(B)** The MC252 oil underwent phase separation from the Hanks Balanced Salt Solution (HBSS) following extensive mixing and then resting on the bench for 1 hour. The saline accommodated phase of the mixture was used to expose the mice in these studies. The other treatments of Corexit 9500 and 9527 solutions alone and in combination with MC252 oil mixed well with the HBSS solution without phase separation (not shown).

Supplemental Figure 2

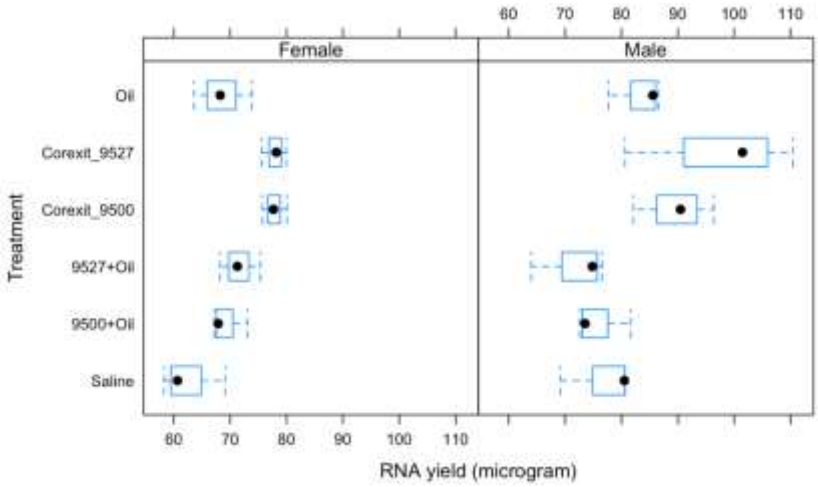

Supplemental Figure 2. RNA yield from treated mice. Total RNA yield recovered from the left lung of female mice was 60-80μg while that from male mice was higher at 75-100μg per left lung before DNase treatment. Shown is the RNA yield from different treatment groups in both sexes. Using multiple regression analysis to model RNA yield as a dependent variable and treatment, sex and weight as independent variables, it was found that male mice had significantly higher RNA yield than female mice ($p = 1.42e-3$). Compared with saline (control) treatment, mice with Corexit 9527 and Corexit 9500 treatments also had a significantly higher RNA yield (with p values of 7.2e-5 and 1.59e-3, respectively). Weight was not a significant predictor for RNA yield.

Supplemental figure 3.

A.

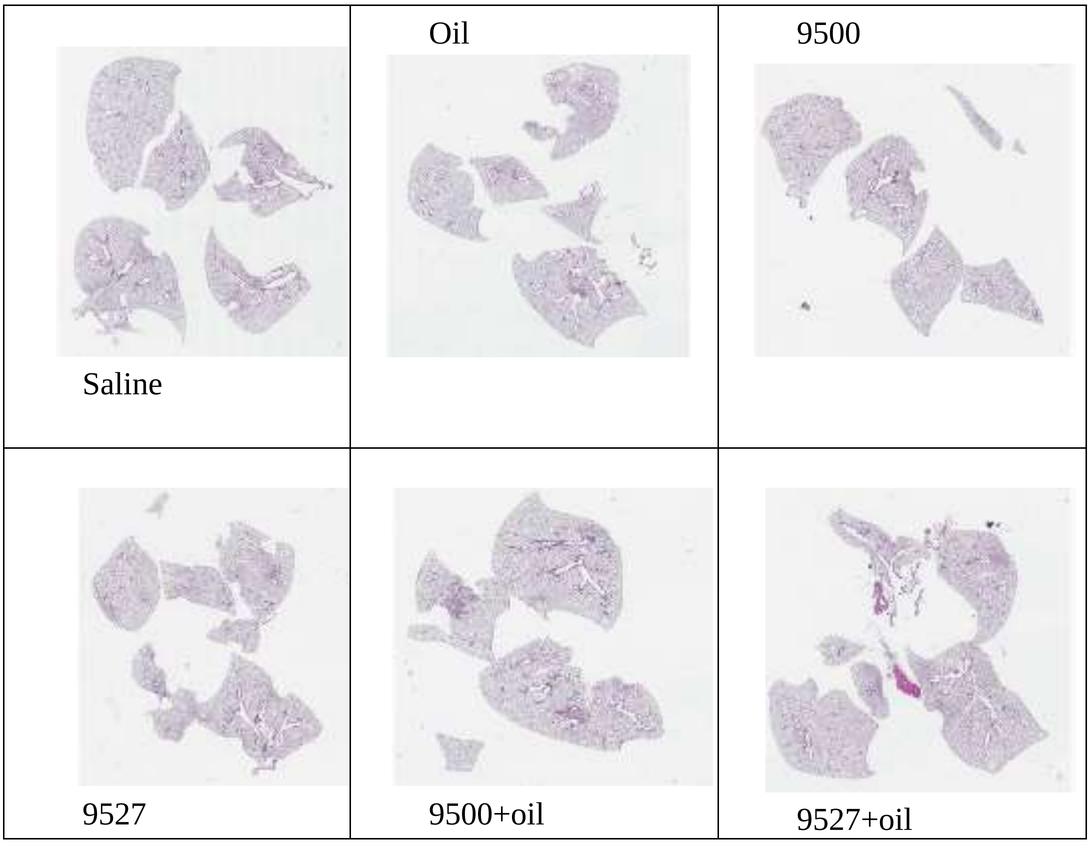

B.

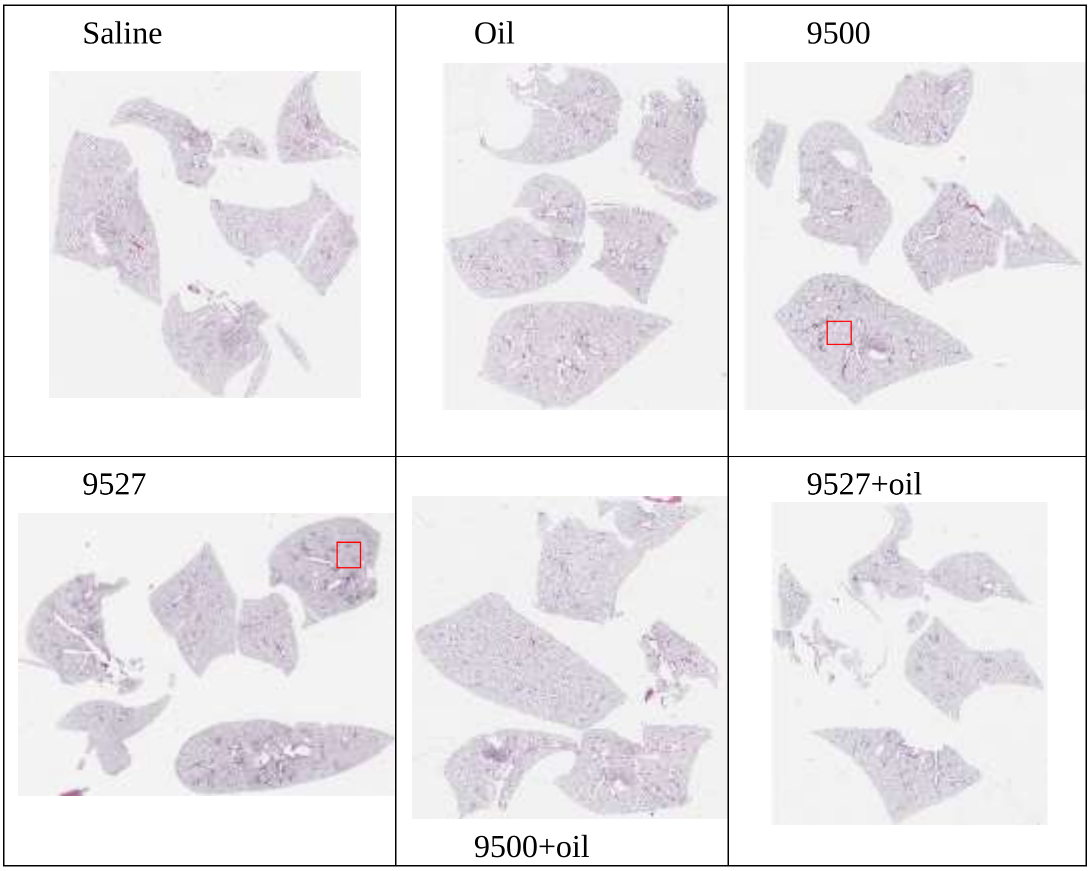

C.

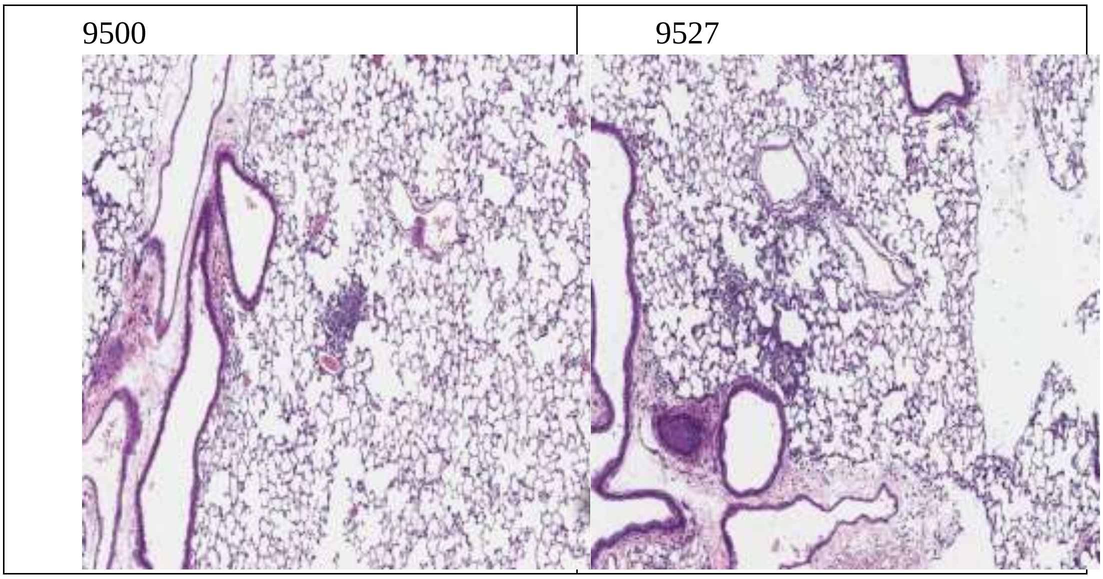

D.

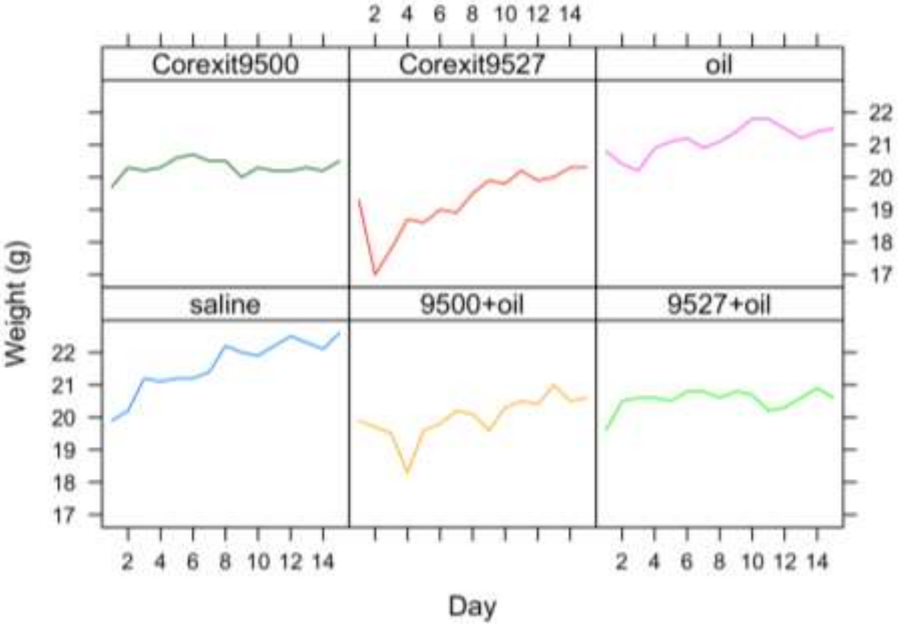

Supplemental Figure 3. Pathological changes associated with exposure of mice to oil and/or dispersant in preliminary experiments. (A) Whole slide images of lung tissue sections from female mice exposed to 0.1 SAF in preliminary experiment 2. **(B)** Same as part A with male mice (preliminary experiment 3). See panel C for a higher power image of the boxed area. **(C)** Higher power images of lung tissue sections in the boxed area of panel B showing minor focal inflammation areas in male mice exposed to Corexit 9527 or Corexit 9500 in preliminary experiment 3. **(D)** Weight change of mice in preliminary experiment 2.

Supplmental Figure 4

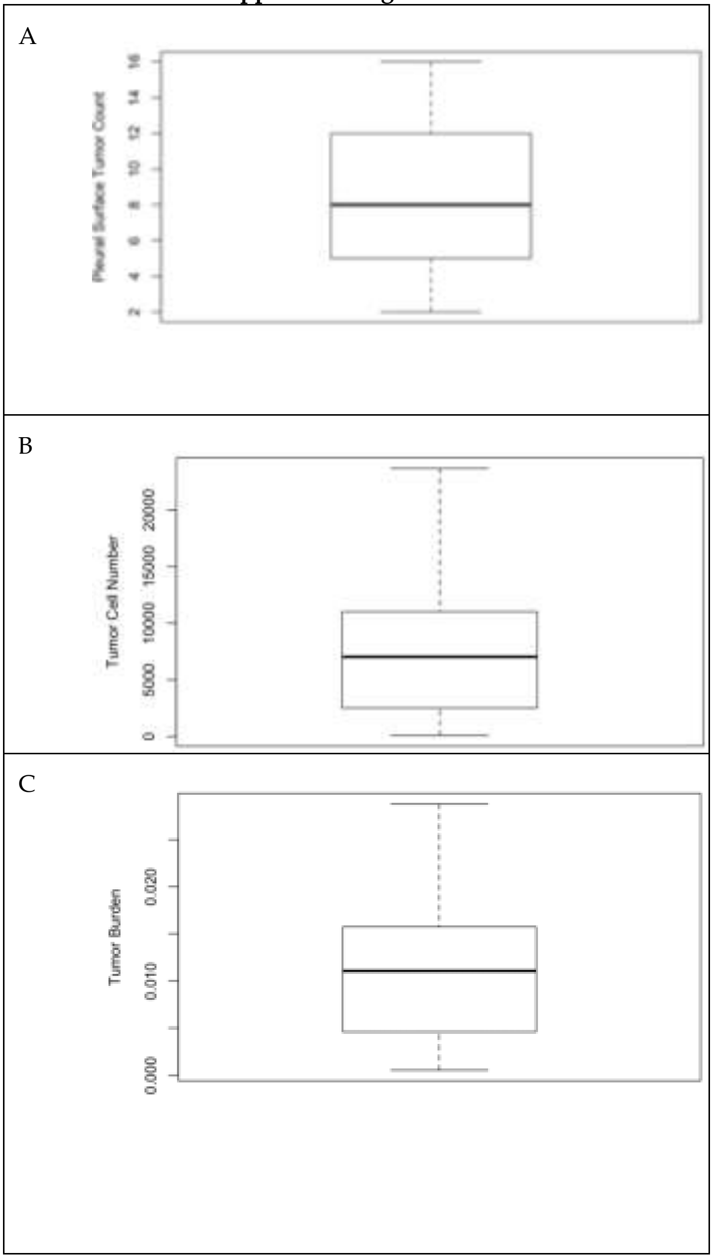

Supplemental Figure 4. Box plots for the K-Ras^{LA1} mice lung tumor indices. (A) Box plot for pleural surface tumor count; **(B)** Box plot for tumor cell number; **(C)** Box plot for tumor burden.

Supplemental Figure 5

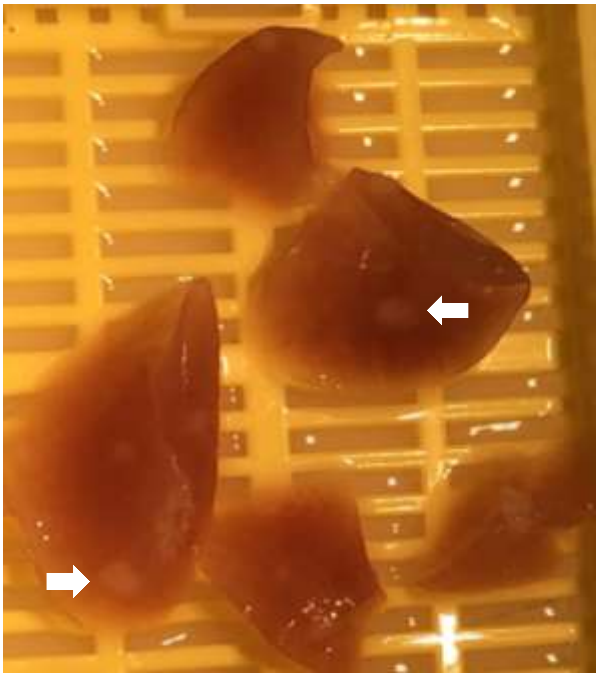

Supplemental Figure 5. Representative tumor nodules on the pleural surface of a K-Ras^{LA1} mouse. Fixed lung from a K- Ras^{LA-1} mouse prior to paraffin embedding is shown. Multiple tumor nodules on the pleural surface appear as small white areas. Two tumor nodules are indicated by arrows.

Supplemental Figure 6

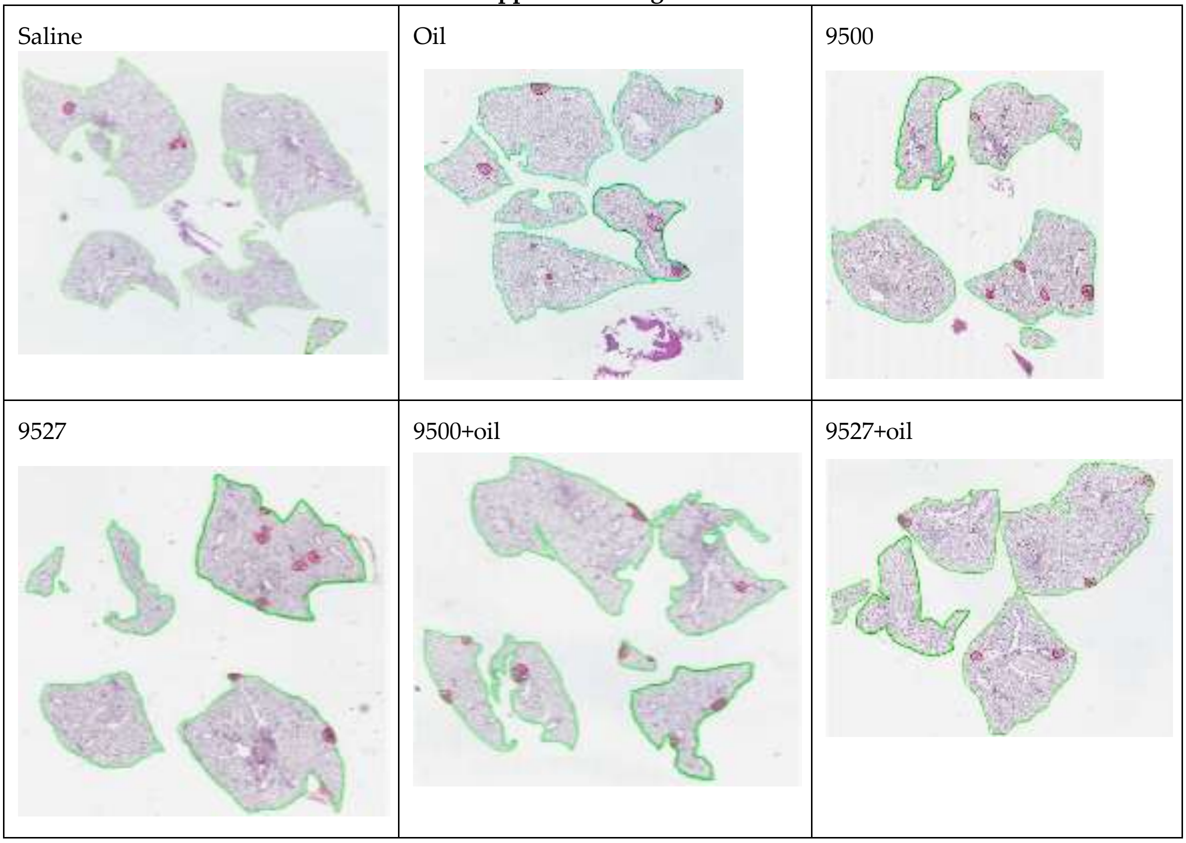

Supplemental Figure 6. Tumor histopathology of K-Ras^{LA1} mice. Whole slide images of lung tissue sections from K-Ras^{LA1} mice with a representative example from a mouse after each treatment. The ratio of the areas of

lung tumor (encircled red) to whole lung areas (encircled green) were determined by measurement with the slide scanner to calculate the tumor burden.

Supplemental Table 1: Primers for qRT-PCR experiments

Genes	Forward primer	Reverse primer
Clspn	ACGGAAATGAGTTATCCAAAGGC	AGTGCAGATTCTCGGACCAGA
Ticrr	GCCTGCTGTCACAAAGTGATG	GCCCCCTGAGAGTCGAAGA
Chek1	GTTAAGCCACGAGAATGTAGTGA	GATACTGGATATGGCCTTCCCT
Fen1	TTCACGGCCTTGCCAAACTAA	ACAGCAATCAGGAACTGGTAGA
Rad51ap1	GTGCGTCCTACCAGAAATAGAAA	CCGCTTTTGACTTGTTTACAGGT
Dtl	ACGCAAGGCAGAAAATTCATCC	GAGCTGGGAGTAATGGTGACC
PCLAF	ACCAAAGCAAACTACGTTCCA	TTTTCCCGACGAACTTGAAGAA
Uhrf1	CCACACCGTGAACTCTCTGTC	GGCGCACATCATAATCGAAGA
Chaf1b	TTCACGACGACAGCATGAAGT	TGTCACATTCTCACCAGATTCCA
Bard1	AAGGAGCCCGTGTGCTTAG	TTGCCCTAGATGTGTTGTCTTTT
Chek2	TGACAGTGCTTCCTGTTCACA	GAGCTGGACGAACCCTGATA

Supplemental Table 2. Number of differentially expressed genes for different sexes and treatments.

Sex and treatments	# of Upregulated Genes	# of Downregulated Genes	Total # of differentially expressed genes
All subjects, Oil	213	340	553
All subjects, Corexit 9500	588	946	1,534
All subjects, Corexit 9527	707	519	1,226
All subjects, 9500+oil	164	381	545
All subjects, 9527+oil	277	958	1,235
Male, Oil	917	515	1432
Male, Corexit 9500	563	367	930
Male, Corexit 9527	1,384	1,123	2,507
Male, 9500+oil	752	665	1417
Male, 9527+oil	627	461	1088
Female, Oil	158	300	458
Female, Corexit 9500	316	832	1,148
Female, Corexit 9527	320	230	550
Female, 9500+oil	88	246	334
Female, 9527+oil	88	880	968
